# Factors influencing late HIV presentation in China: results from logistic regression and Bayesian network analyses

**DOI:** 10.1186/s12879-025-12429-6

**Published:** 2026-01-16

**Authors:** He-he Zhao, Dong-hang Luo, Li-ping Fei, Shi Wang, Fang-fang Chen, Qian-qian Qin, Chang Cai, Yi-Chen Jin, Jie Xu, Hou-lin Tang, Fan Lyu

**Affiliations:** 1 National Center for AIDS/STD Control and Prevention, Chinese Center for Disease Control and Prevention, Beijing, 102206 People’s Republic of China; 2 National Key Laboratory of Intelligent Tracking and Forecasting for Infectious Disease, Beijing, 102206 People’s Republic of China

**Keywords:** HIV, Factors, Late presentation (LP), Logistic regression, Bayesian network

## Abstract

**Background:**

Late presentation (LP) of HIV infection remains a major challenge to epidemic control, leading to advanced immunodeficiency, poorer treatment outcomes, and ongoing transmission before diagnosis. Despite expanded testing and awareness efforts, a considerable proportion of people living with HIV (PLHIV) in China are still diagnosed late.

**Methods:**

This study analyzed 386,704 newly reported HIV cases (2019–2022) from the National HIV/AIDS Comprehensive Response Information Management System (CRIMS). Logistic regression was used to identify significant predictors of LP, and a Bayesian network was constructed to model the complex interrelationships among variables.

**Results:**

Logistic regression identified several factors associated with LP of HIV. Key factors included being male (aOR = 1.3), over 60 (aOR = 3.36), Han ethnicity (aOR = 1.16), education at below senior high school (aOR = 1.1), being a farmer or worker (OR = 1.04), transient population (aOR = 1.18), engaging in homosexual transmission (aOR = 1.1), being examined at other institutions (hospitals) (aOR = 1.27), having non-marital partners(lifetime history), and a history of STDs (aOR = 1.03) (*P* < 0.05). Bayesian network analysis revealed that age, gender, and sample sources were the key factors associated with LP of HIV. Among them, age played a central role in the model, directly influencing occupation, transmission routes, education level, transient status, and non-marital partners. Ethnicity indirectly affected LP through occupation, while education influenced LP indirectly by shaping occupation, non-marital partners, and transient status. In addition, a history of STDs not only directly affected sample sources but also indirectly increased the risk of LP through transient population status.

**Conclusion:**

This mixed-model approach demonstrated that demographic, behavioral, and structural factors jointly contribute to LP in China through complex associative pathways. Integrating logistic and Bayesian frameworks provides a more comprehensive understanding of HIV diagnostic delays, informing precision-targeted testing and intervention strategies.

**Supplementary Information:**

The online version contains supplementary material available at 10.1186/s12879-025-12429-6.

## Introduction

Despite significant advances in the prevention and treatment of HIV/AIDS, late presentation (LP) of HIV remains a serious challenge in many countries [1, 2]. Late HIV detection leads to missed opportunities for timely intervention, resulting in increased morbidity and mortality among affected individuals [3]. Furthermore, LP exacerbates the risk of HIV transmission within communities, posing a public health concern that necessitates immediate attention [4]. Therefore, the accurate prediction and timely diagnosis of LP cases are crucial for improving patient outcomes and controlling the spread of the virus. Recent years have witnessed the rapid evolution of data science and machine learning technologies, prompting researchers to explore advanced statistical methodologies for the analysis and prediction of LP [5, 6]. Among these, logistic regression and Bayesian networks have emerged as two powerful statistical tools, each offering distinct advantages in HIV research [7]. Logistic regression, a traditional statistical method, effectively identifies independent risk factors influencing HIV LP. For example, research has demonstrated that variables such as age, education level, and frequency of HIV testing are significant determinants of LP [8, 9]. Conversely, Bayesian networks, as an innovative probabilistic graphical model, excel in capturing complex interactions among variables and yielding intuitive visual representations [10]. Miranda et al. [11] employed Bayesian networks to analyze determinants of LP in Europe, uncovering direct relationships involving viral load, transmission patterns, age, and time of infection, as well as potential indirect influences. This multifaceted approach offers valuable insights for developing targeted intervention strategies.

Logistic regression may fall short in fully capturing the intricate relationships among variables, while Bayesian networks can encounter computational complexities when managing numerous variables. Integrating logistic regression with Bayesian networks allows simultaneous quantification of direct effects and exploration of complex interdependencies, offering a more holistic understanding of HIV LP determinants. Emerging research supports the effectiveness of mixed models in enhancing predictive performance across various medical domains. For instance, Chu et al. (2022) successfully combined logistic regression and Bayesian networks in cancer prognosis prediction, demonstrating superior predictive capabilities compared to the use of a single method [12]. This success serves as a noteworthy precedent for the application of similar strategies within HIV research. Moreover, the landscape of HIV epidemiology is evolving, particularly with the introduction of innovative prevention strategies such as Pre-Exposure Prophylaxis (PrEP). Research by Zhou et al. (2022) utilizing Bayesian networks to analyze factors influencing PrEP acceptance has provided new insights into how these preventive measures impact HIV testing and diagnostic patterns [7]. This underscores the need for dynamic and responsive predictive models that can adapt to changing epidemiological trends.

Considering the above background, this study aims to investigate the factors influencing LP of HIV and, by incorporating Bayesian network methods, to further elucidate the complex interactions and potential risk pathways among these factors, thereby providing a more comprehensive understanding of the mechanisms underlying LP.

## Methods

### Source of data

Data were extracted from the National HIV/AIDS Comprehensive Response Information Management System (CRIMS) database, a nationwide database that compiles comprehensive records on HIV/AIDS diagnosis, treatment, and follow-up from 31 provinces in China. The study included newly reported HIV cases from 2019 to 2022 that had available CD4 count data or documentation of an AIDS-defining event. Exclusion criteria: (1) missing or incomplete CD4 data; (2) inconsistent or implausible demographic/clinic records; (3) duplicate entries. This study collected 520,044 newly reported cases from January 2019 to December 2022. After excluding 133,340 cases with missing, duplicated or incomplete data, 386,704 cases remained, of which 256,854 were classified as LPs and 129,850 as non-late presentations (NLPs), used for further analysis (Supplementary Fig. 1).

### Relevant definitions

**Late Presentation (LP) of HIV** is defined as either: (a) CD4 count below 350 cells/mm³ at diagnosis, or (b) the presence of an AIDS-defining event, regardless of the CD4 count. Samples lacking a reported initial CD4 count, or those with an initial CD4 count recorded outside the period from 2019 to 2022, were not included in this analysis. **Transient population** is commonly used to describe the transient population in China, particularly referring to individuals who leave their place of habitual residence for work or other reasons but do not settle permanently in their new location. Transient status is a defining characteristic of the migrant population under China’s hukou system.

### Statistics

Categorical variables were reported as number of relevant cases and percentages. Multifactor logistic analysis was conducted using the *autoReg* package in R 4.4.1, with a significance level set at α = 0.05. The *bnlearn* package was used for Bayesian network structure learning on significant factors, employing the Tabu search algorithm. *Netica* software [13] was used to visualize the Bayesian networks.

## Results

### Domestic analyses and screening of factors influencing LP

This study investigated the factors influencing LP of HIV using univariate and multivariate logistic regression analyses. The results indicated that several factors, including gender, age, ethnic groups, education level, occupation, transient status, transmission routes, sample sources, non-marital sexual partners, and history of STDs, are associated with the likelihood of LP (Table 1). Specifically, the LP rate among males (66.8%) was higher than that among females (65.0%), with an adjusted odds ratio (aOR) of 1.30. Age emerged as a critical determinant, with individuals aged 60 years or above exhibiting the highest LP rate (75.8%) and an aOR of 3.36. Education level may be another important factor: individuals with a high school education or below had an LP rate of 69.7% and an aOR of 1.10. Similarly, farmers and workers had a higher LP rate (69.9%) with an aOR of 1.04. Transient population also showed an elevated LP rate (65.9%) with an aOR of 1.18. The analysis of sample sources showed that the LP rates at STD clinics (60.3%) and testing consultations (59.8%) are lower than those from other sources (69.3%, mainly indicate hospital). Moreover, those reporting multiple non-marital sexual partners exhibited a higher LP rate (70.2%), whereas individuals with unspecified numbers of non-marital partners had a comparatively lower LP rate (59.6%), with an aOR of 0.91.

**Table 1:** Univariate/Multivariate logistic regression analysis of factors affecting LP of HIV

Variable	Total(n=386,704)	Late presentation (LPs)(n=256,854) (%)	OR (95%CI)	aOR (95% CI)
Gender				
Female	86,031(22.2)	55,879 (65.0)	Ref.	Ref.
Male	300,673(77.8)	200,975 (66.8)	1.09 (1.07-1.11)^*^	1.30 (1.28-1.3)^*^
Age				
0-19	11,573(3.0)	4,977 (43.0)	Ref.	Ref.
20-39	133,253(34.5)	76,264 (57.2)	1.77(1.71-1.84)^*^	1.66 (1.60-1.73)^*^
40-59	151,091(39.1)	106,843 (70.7)	3.20 (3.08-3.33)^*^	2.75 (2.64-2.87)^*^
≥ 60	90,787(23.5)	68,770 (75.8)	4.14 (3.98-4.31)^*^	3.36 (3.22-3.50)^*^
Transient population				
No	242,421(62.7)	161,804 (66.8)	Ref.	Ref.
Yes	144,283(37.3)	95,050 (65.9)	0.96(0.95-0.98)^*^	1.18(1.11-1.15)^*^
Farmers or workers				
No	170,017(44.0)	105,439 (62.0)	Ref.	Ref.
Yes	216,687(56.0)	151,415 (69.9)	1.42 (1.40-1.44)^*^	1.04 (1.03-1.06)^*^
Married with spouse				
No	211,241(54.6)	134,697 (63.8)	Ref.	Ref.
Yes	175,463(45.4)	122,157(69.6)	1.30 (1.28-1.32)^*^	0.99 (0.98-1.01)
Ethnic groups				
Han	323,497(83.7)	217,110(67.1)	Ref.	Ref.
Others	63,207(16.3)	39,744(62.9)	0.83 (0.82-0.84)^*^	0.86(0.85-0.88)^*^
Below senior high school				
No	119,197(30.8)	70,390(59.1)	Ref.	Ref.
Yes	267,507(69.2)	186,464(69.7)	1.60 (1.57-1.62)^*^	1.10 (1.08-1.12)^*^
Homosexual transmission				
No	285,088(73.7)	197,752(69.4)	Ref.	Ref.
Yes	101,616(26.3)	59,102(58.2)	0.61 (0.60-0.62)^*^	0.91 (0.88-0.94)^*^
Sample sources				
Others	268,371(69.4)	186,035(69.3)	Ref.	Ref.
STD clinic	23,527(6.1)	14,172(60.3)	0.67 (0.65-0.69)^*^	0.82 (0.80-0.85)^*^
Testing consulting	94,806(24.5)	56,647(59.8)	0.66 (0.65-0.67)^*^	0.79 (0.77-0.80)^*^
Non-marital sexual partners^#^				
>1	159,741(41.3)	112083(70.2)	Ref.	Ref.
≤ 1	99,891(25.8)	68994(69.1)	0.95 (0.93-0.97)^*^	0.98 (0.97-1.00)
unspecified	127,072(32.9)	75777(59.6)	0.63(0.62-0.64)^*^	0.91(0.89-0.93)^*^
With STD^#^				
No	349,861(90.5)	232,734(66.5)	Ref.	Ref
Yes	36,843(9.5)	24,120(65.5)	0.95 (0.93-0.98)^*^	1.03(1.01-1.05)^*^

Note: * *P* < 0.05, statistically significant

CI: confidence interval

Non-marital sexual partners: the cumulative lifetime history of sexual partners as self-reported during case interviews

With STD: including syphilis, gonorrhea, genital herpes, and genital warts

### Construction of Bayesian network

**Fig. 1:**
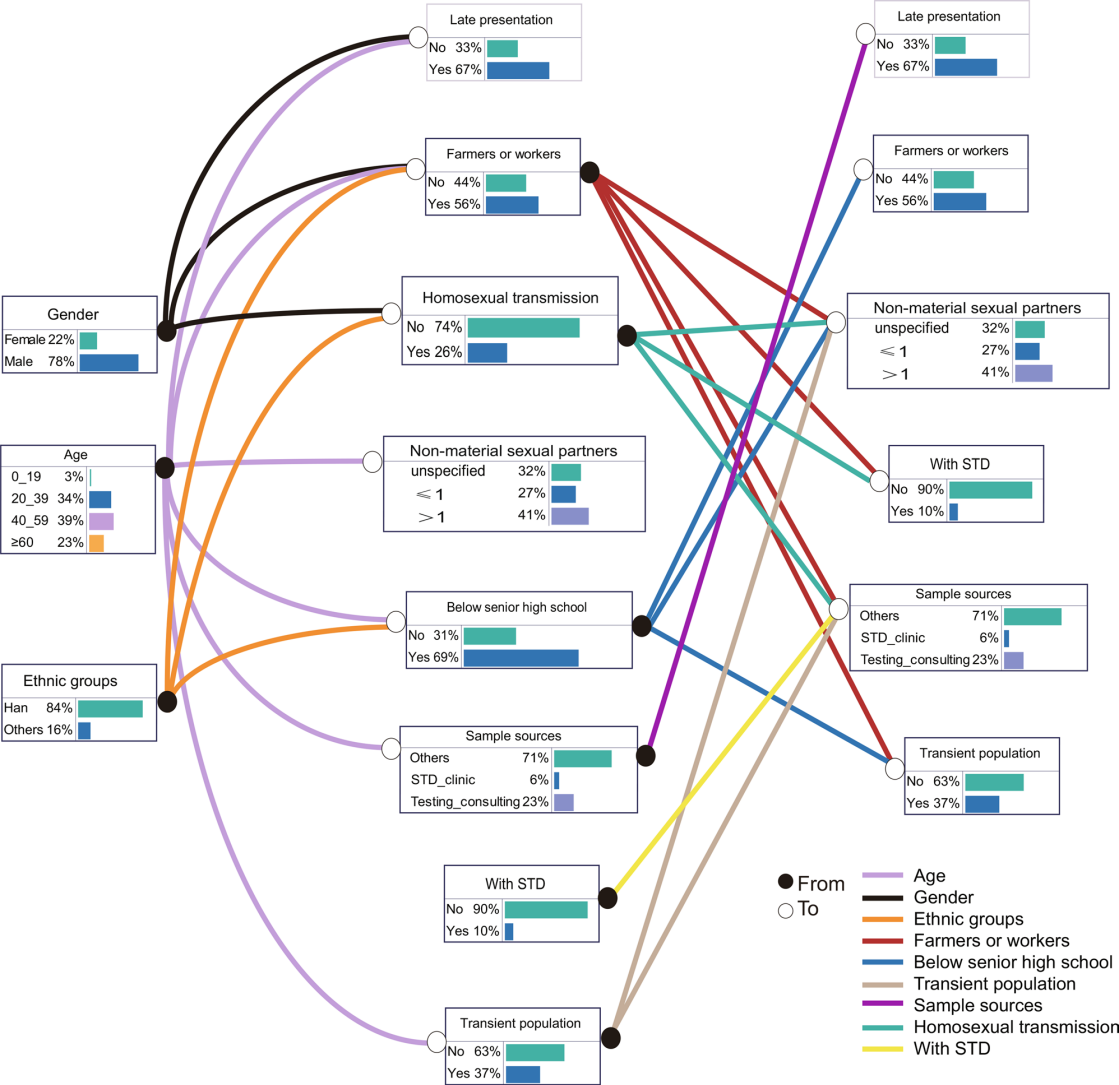
Bayesian network of factors influencing HIV late presentation constructed using the MMHC algorithm

Based on the 10 statistically significant variables identified through multiple logistic regression analysis, we constructed a Bayesian network with 11 nodes and 25 directed edges related to factors influencing LP, obtaining the conditional probabilities for each node (Supplementary Table 1). The results showed that age, gender, and sample sources were directly related to LP of HIV, while occupation, transient population, homosexual transmission and history of STDs were indirectly related to LP of HIV by affecting the sample sources (Fig. 1). The Bayesian network described the complex relationships among these factors. Specifically, age was associated with occupation, education level, transient population, and non-marital sexual partners, and older age was likely correlated with lower education levels and a higher proportion of farmers and workers. Ethnic groups and gender directly influenced occupation and transmission routes, with Han males being more likely to work as farmers and workers, primarily engaging in heterosexual transmission. Education level directly affected occupation, transient population, and non-marital sexual partners. Occupation and transmission routes were related to transient population, sample sources, non-marital sexual partners, and the history of STDs, while STD history only directly affects sample sources.

### Predictive inference for LP

**Fig. 2:**
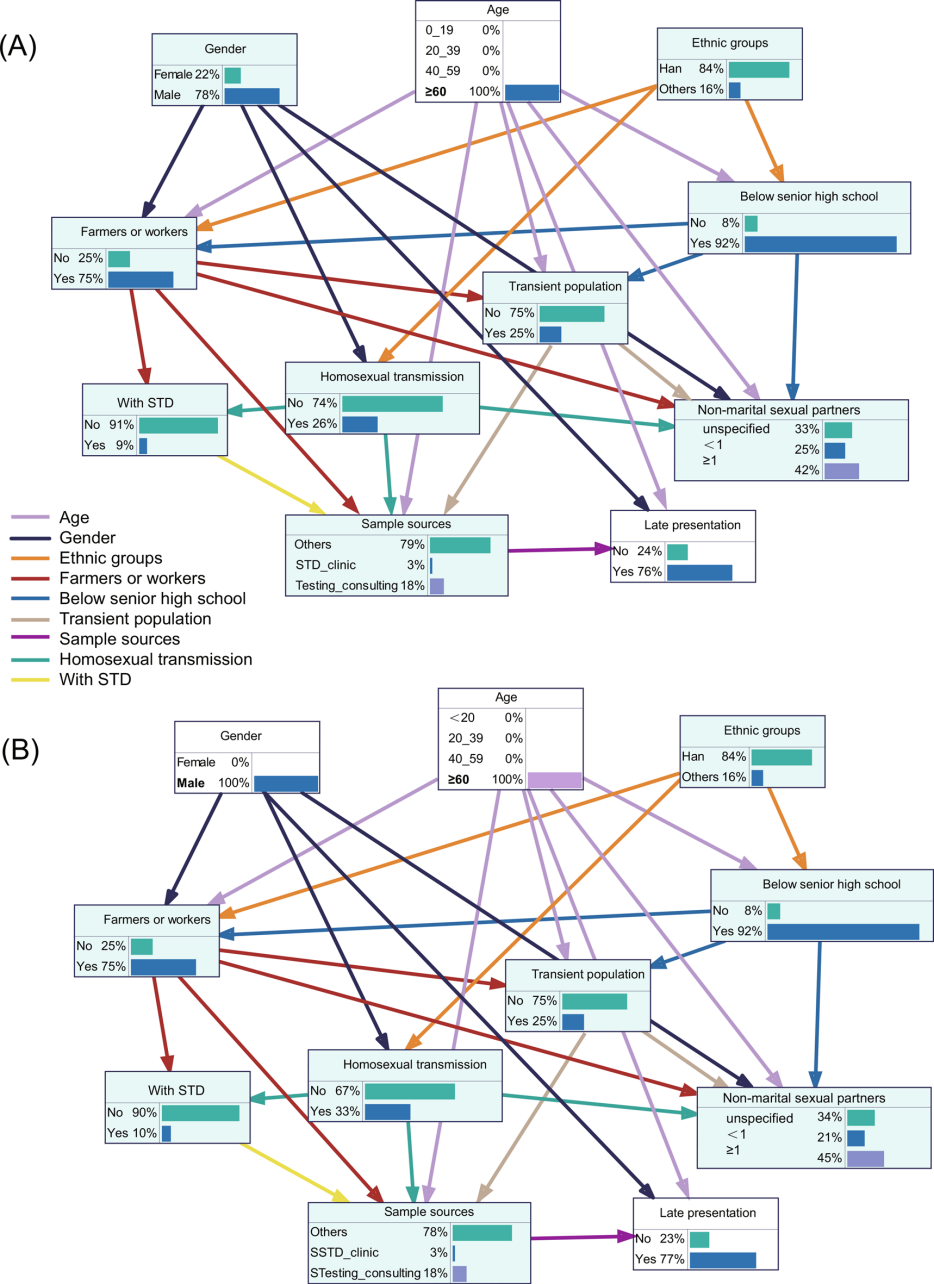
Risk inference of HIV late presentation. (**A**) Risk inference for late presentation among HIV-infected individuals over 60 years old; (**B**) Risk inference for late presentation among male HIV-infected individuals over 60 years old

Predictive reasoning leverages the prior probabilities of nodes and their connections to estimate outcome probabilities. By adjusting the probability distribution of specific factors, we observed changes in outcome probabilities under different influencing factors, allowing us to identify high-risk populations. To enhance the predictive power of the model and more accurately pinpoint high-risk factors, we further restricted age (Fig. 2A) and both age and gender (Fig. 2B). For individuals over 60 years old, the probability of LP increased from 67% to 76% (Fig. 2A). When considering only males in this age group, the probability rose slightly to 77% (Fig. 2B). This age restriction indicates that older adults are more likely to be diagnosed at a late stage of the disease. Additionally, comparing the two Bayesian networks showed that adding the gender restriction increased the proportion of male homosexual transmission from 26% to 33%, suggesting that homosexual transmission routes may be more significant among elderly males. Co-infection rates with STDs also rose, indicating a heightened risk of both homosexual transmission and STD co-infection in men aged 60 and older. Through Bayesian network model analysis, we simulated the probability of LP in 768 hypothetical scenarios, yielding results consistent with logistic regression.

### Probability of relationships among factors influencing LP

**Fig. 3:**
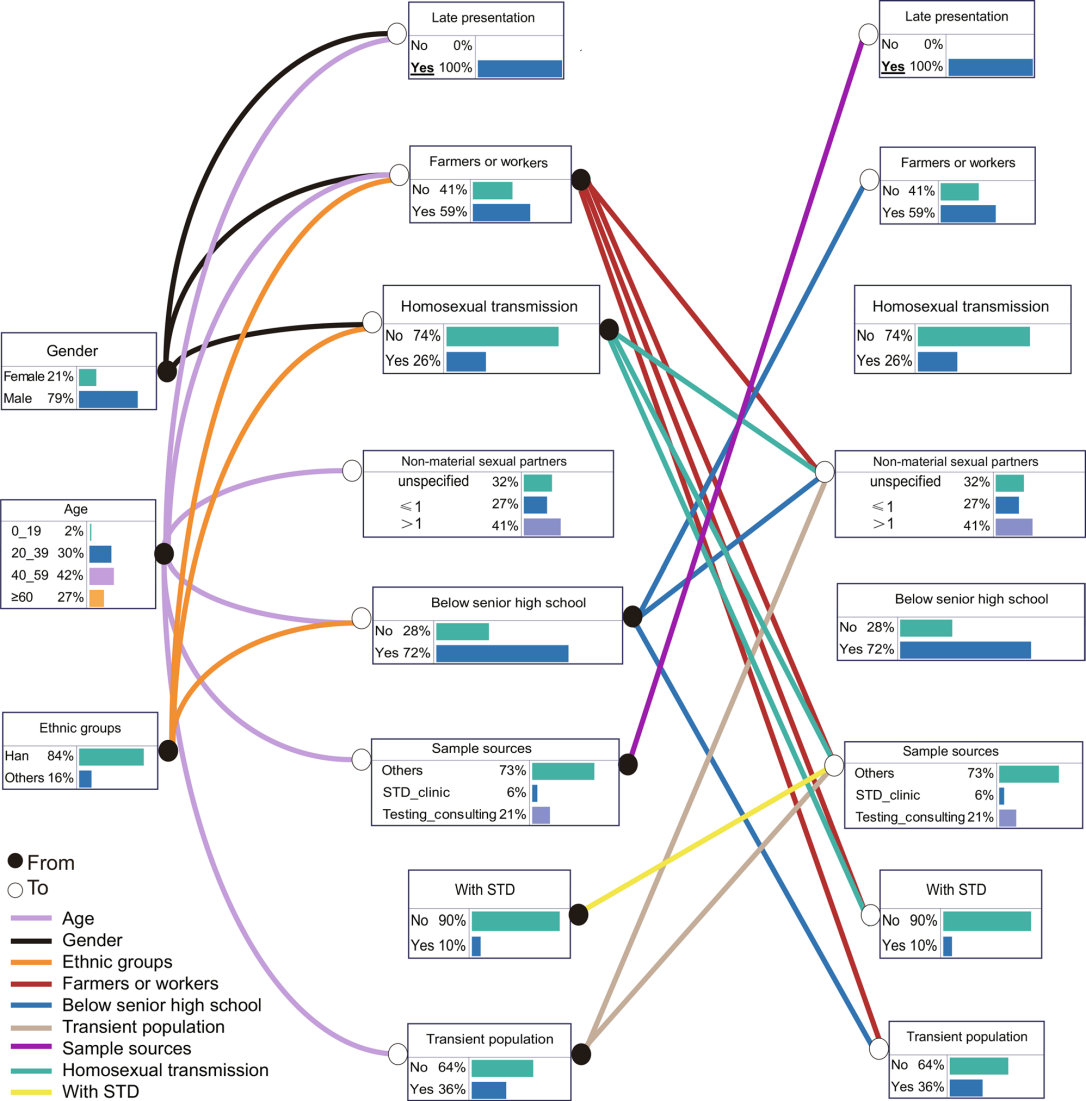
Bayesian network risk inference for HIV late presentation

The results of Bayesian network indicated that age, gender, and sample sources directly influence the probability of LP, while education level, occupation, ethnic groups, and transient population status indirectly influence LP risk by impacting those direct factors (Fig. 3). Setting the probability of LP to 100% revealed key influencing factors: the proportion of individuals over 40 years old increased from 62% to 69%, and the number of farmers or workers rose from 56% to 59%. The proportion of individuals with education below senior high school increased from 69% to 72%, while variables such as homosexual transmission and non-marital sexual partners remained unchanged, and the transient population decreased from 37% to 36% (Fig. 1 vs. Fig. 3). Additionally, transmission routes, including homosexual transmission, and co-infection with STDs may indirectly influence LP through complex mediation effects. Among males, the proportion of transmission via homosexual routes was higher, and those co-infected with STDs had an increased probability of LP. Overall, Bayesian network analysis identified being male, middle-aged or older, having a low education level, working as farmers or laborers, having a history of STDs, and having multiple non-marital sexual partners as the factors influencing LP. These factors contribute to the risk of HIV LP through complex associative pathways.

## Discussion

The high proportion of LP HIV cases (66.4% of total cases) highlights a significant public health challenge in detection and early intervention. LP hinders prevention and effective treatment of HIV infection. In this study, we found that age is a key factor of LP, with risk increasing as age advances. Older individuals (60 or above) are at increased risk, represent a growing proportion of LPs, and may require interventions tailored to different age group [14]. The higher percentage of males among LPs(76.8%) aligns with recent research [15]. An Asian study also found that approximately 72% of LPs were male, supporting the trend of male predominance in late-stage HIV presentations [16]. This may be attributed to differences in healthcare-seeking behaviors, as men are generally less likely to engage in routine health surveillance. We observed that individuals diagnosed at STD clinics and through consulting and testing have a lower aOR of LP compared to those diagnosed in other sources (hospitals). The WHO emphasizes that expanding community-based testing, including HIV self-testing, is essential for enhancing global HIV prevention efforts [17]. Providing diverse HIV testing methods is an effective strategy to achieve detection, prevention, treatment and to maintain a low incidence rate [18]. Countries should expand the use of HIV self-testing, integrate it with other health services (such as STD diagnosis), and promote social network testing approaches to address coverage gaps caused by access, stigma, lack of awareness, or economic conditions [19]. The lack of significant difference in STD history between LPs and NLPs is noteworthy, contrasting with earlier assumptions that prior STD diagnosis might lead to earlier HIV detection. A recent study by Shi L, et al. in China also found that STD history was not significantly associated with LP of HIV [20], suggesting that current STD services may not effectively leverage opportunities for early HIV detection.

Using logistic regression and Bayesian network analysis provides a comprehensive framework for understanding the factors influencing HIV LP, which is crucial for developing targeted interventions. This study identified 10 factors—gender, age, ethnic groups, transient population status, occupation, non-marital sexual partners, education, transmission routes, sample sources, and STD history—aligning with recent literature that emphasizes the multifactorial nature of HIV LP [21, 22]. Older adults often experience delayed testing due to lower perceived HIV risk and limited awareness among healthcare providers [23]. The higher proportion of LP among farmers and workers (75%) suggests socioeconomic barriers, including restricted access to healthcare and testing services [24].These challenges are particularly pronounced in rural areas, where targeted outreach initiatives and mobile testing units can play a pivotal role in reducing delayed diagnosis [25]. Additionally, limited health literacy among individuals with lower education level, who account for the majority of LPs, impairs awareness of HIV risk and the importance of timely testing [26]. Tailored public health campaigns employing accessible language and culturally appropriated messaging are essential to mitigate these challenges [27]. Integrating HIV and STD testing within routine healthcare settings could facilitate earlier detection among high-risk populations. Furthermore, the finding that 79% were diagnosed through non-traditional channels highlights a significant gap in the healthcare system’s capacity to engage and retain at-risk individuals. Strengthening formal healthcare networks and improving referral systems are crucial for enhancing early diagnosis rates [28].

Integrating multiple logistic regression and Bayesian network analysis can elucidate the complex mechanisms behind HIV LP, laying the groundwork for effective policy formulation and intervention measures. This study revealed critical demographic and behavioral factors associated with LP of HIV, highlighting the interplay between socioeconomic and behavioral determinants that affect healthcare access and disease progression. Research showed that there were complex interactions among the various factors influencing LP, which has drawn extensive attention across multiple disciplines [21]. For example, occupation and education level are closely linked to individuals’ health knowledge and access to sample resources, and these variables may further affect the timing of HIV testing and the risk of late diagnosis through mediating factors such as gender and marital status [29]. Transient populations are generally more likely to be diagnosed with HIV LP due to limited access to medical services and lack of social support, with this effect often moderated by age and transmission routes [30]. A history of STDs serves as an important covariate, as it not only indicates an increased risk of HIV infection but may also be related to the source of testing, such as voluntary counseling and testing or hospital inpatient screenings, which can affect diagnosis timing [31].

The complex associations between transmission routes and factors like gender and marital status also vary across cultural and social contexts. For example, men are more likely to receive late-stage diagnoses in heterosexual transmissions, while women more frequently experience late-stage diagnoses through mother-to-child transmission [32]. This phenomenon may be influenced by education level and health knowledge. Further studies suggest that structural factors, including healthcare system coverage and socioeconomic inequalities, may amplify the interactions among these factors by shaping individual behavior and social environments [33, 34]. Bayesian networks revealed potential conditional dependencies among variables, such as how education level influences health behavior through occupation, ultimately affecting early disease diagnosis. Logistic regression confirmed the independent contribution rates of these factors and highlighted the importance of multifactorial interactions in specific groups. These findings underscore the need for a comprehensive public health approach to tackle structural, behavioral, and systemic barriers to early HIV diagnosis. Recommended measures include strengthening national policies for routine HIV testing in high-risk groups, developing gender- and occupation-specific health promotion activities to reduce stigma, integrating HIV testing into routine healthcare visits, enhancing health literacy programs for low-education populations, and improving healthcare accessibility for migrant workers and marginalized communities.

This study analyzed various factors influencing LP of HIV in China using logistic regression and Bayesian network methods, but it has several limitations. First, the data from CRIMS, despite a large sample size of 520,044 cases, may exhibit selection bias, particularly concerning specific populations like transient individuals and those with low educational levels. Second, while multiple statistical methods were used, the logistic regression model might not fully capture the complex relationships among variables, and Bayesian networks could face computational challenges with many variables. Additionally, the study focused mainly on quantitative analysis and lacked a deep qualitative exploration of the sociocultural factors influencing HIV late presentation, potentially limiting the understanding of these factors. Finally, the cross-sectional design restricts the clarity of causal relationships, future research should consider a longitudinal approach to better understand the temporal factors affecting HIV LP. These limitations highlight the need for cautious interpretation of the findings and suggest areas for improvement in future studies.

## Conclusion

Our findings revealed that late HIV presentation in China may be shaped by a complex interplay of demographic, behavioral, and structural determinants. By integrating logistic regression and Bayesian network analyses, this study uncovered both direct and indirect pathways influencing LP, with age, gender, and sample sources acting as primary determinants, while education level, occupation, ethnicity, and migrant status exert indirect effects through these key variables.

## Supplementary Information

Below is the link to the electronic supplementary material.

Supplementary Material 1

Supplementary Material 2

Supplementary Material 3

## Data Availability

The data underlying the graphs are presented in the main figures. Materials related to the figures are available from Fan Lyu upon reasonable request.

## Abbreviations

HIV: Human immunodeficiency virus
LP: Late presentation
NLP: Non-late presentation
STD: sexually transmitted disease
PrEP: Pre-Exposure Prophylaxis
aOR: Adjusted odds ratio
CI: Confidence Interval
