# Supplementary material for: Factors influencing late HIV presentation in China: results from logistic regression and Bayesian network analyses
Source: BMC Infect Dis. 2026 Jan 16;26:308. doi: 10.1186/s12879-025-12429-6 (PMC12892577; doi:10.1186/s12879-025-12429-6)
Supplement: Supplementary file 1 — Supplementary Material 1 [file 12879_2025_12429_MOESM1_ESM.docx]

Conditional probability table

| Variable | States | Percent (%) |
| --- | --- | --- |
|  |  |  |
| Age | 0-19 | 1.2 |
|  | 20-39 | 34.4 |
|  | 40-59 | 39.0 |
|  | ≥60 | 23.4 |
| Gender | Male | 77.8 |
|  | Female | 22.2 |
| Ethnic groups | Han | 83.7 |
|  | Others | 16.3 |
| Farmers or workers | Yes | 56.0 |
|  | No | 44.0 |
| Below senior high school | Yes | 69.1 |
|  | No | 30.9 |
| Transient population | Yes | 37.3 |
|  | No | 62.7 |
| Sample sources | Testing consulting | 24.5 |
|  | STD clinic | 6.1 |
|  | Others | 69.3 |
| Homosexual transmission | Yes | 26.3 |
|  | No | 73.7 |
| Non-marital sexual partners | ＞1 | 41.3 |
|  | ≤1 | 25.9 |
|  | unspecified | 32.8 |
| With STD | Yes | 9.6 |
|  | No | 90.4 |
| Late presentation | Yes | 66.4 |
|  | No | 33.6 |
