# Supplementary figures and images for: Factors influencing late HIV presentation in China: results from logistic regression and Bayesian network analyses
Source: BMC Infect Dis. 2026 Jan 16;26:308. doi: 10.1186/s12879-025-12429-6 (PMC12892577; doi:10.1186/s12879-025-12429-6)

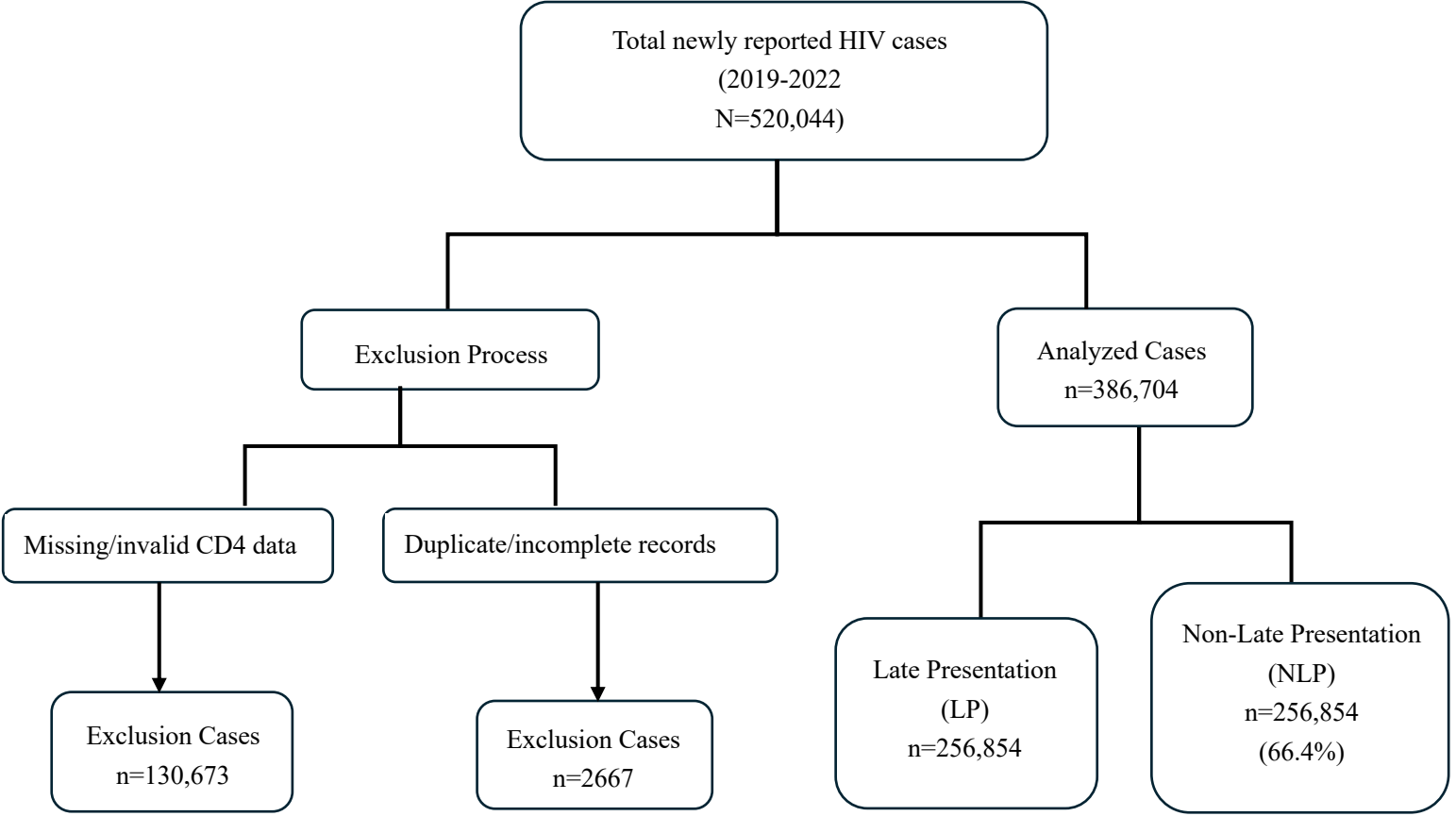

Supplement: Supplementary file 2 — Supplementary Material 2 [file 12879_2025_12429_MOESM2_ESM.pdf]

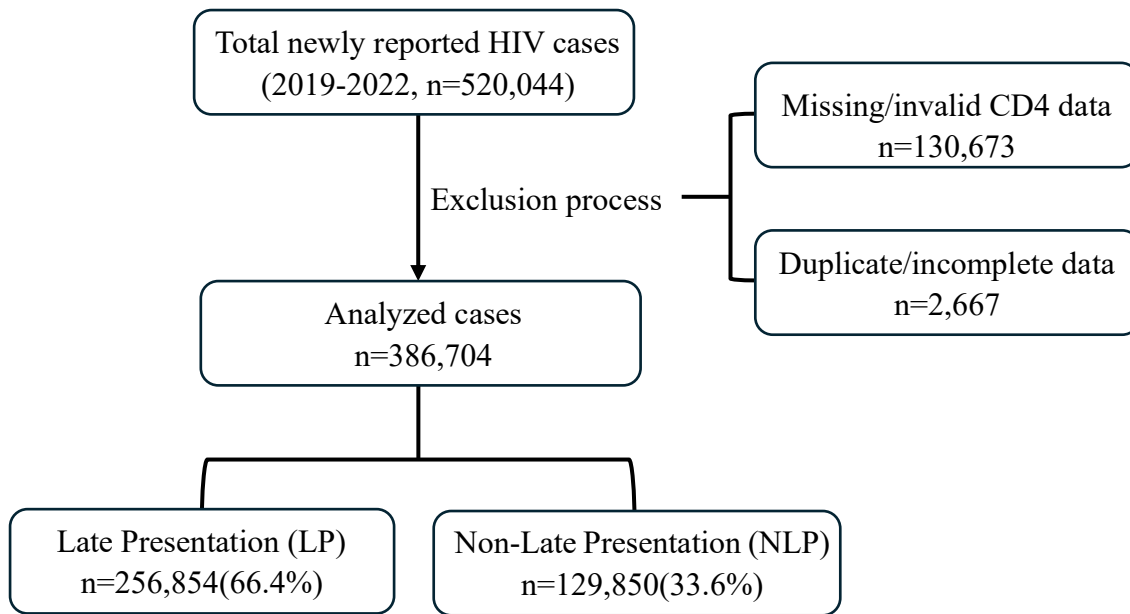

Supplement: Supplementary file 3 — Supplementary Material 3 [file 12879_2025_12429_MOESM3_ESM.pdf]
